# Lignin Metabolism by Selected Fungi and Microbial Consortia for Plant Stimulation: Implications for Biologically Active Humus Genesis

**DOI:** 10.1128/spectrum.02637-22

**Published:** 2022-10-31

**Authors:** Jalil Ur Rehman, Eun-Nam Joe, Ho Young Yoon, Sumin Kwon, Min Seung Oh, Eun Ju Son, Kyoung-Soon Jang, Jong-Rok Jeon

**Affiliations:** a Division of Applied Life Science (BK21Plus), Gyeongsang National University, Jinju, Republic of Korea; b Department of Agricultural Chemistry and Food Science & Technology, Gyeongsang National University, Jinju, Republic of Korea; c IALS, Gyeongsang National University, Jinju, Republic of Korea; d Bio-Chemical Analysis Team, Korea Basic Science Institute, Cheongju, South Korea; University of Minnesota

**Keywords:** plant lignin, environmental microbes, humification, plant stimulation, white-rot fungi

## Abstract

Plant lignin is regarded as an important source for soil humic substances (HSs). Nonetheless, it remains unclear whether microbial metabolism on lignin is related to the genesis of unique HS biological activities (e.g., direct plant stimulation). Here, selected white-rot fungi (i.e., Ganoderma lucidum and Irpex lacteus) and plant litter- or mountain soil-derived microbial consortia were exploited to structurally modify lignin, followed by assessing the plant-stimulatory activity of the lignin-derived products. Parts solubilized by microbial metabolism on lignin were proven to exhibit organic moieties of phenol, carboxylic acid, and aliphatic groups and the enhancement of chromogenic features (i.e., absorbance at 450 nm), total phenolic contents, and radical-scavenging capacities with the cultivation times. In addition, high-resolution mass spectrometry revealed the shift of lignin-like molecules toward those showing either more molar oxygen-to-carbon or more hydrogen-to-carbon ratios. These results support the findings that the microbes involved, solubilize lignin by fragmentation, oxygenation, and/or benzene ring opening. This notion was also substantiated by the detection of related exoenzymes (i.e., peroxidases, copper radical oxidases, and hydrolases) in the selected fungal cultures, while the consortia treated with antibacterial agents showed that the fungal community is a sufficient condition to induce the lignin biotransformation. Major families of fungi (e.g., *Nectriaceae*, *Hypocreaceae*, and *Saccharomycodaceae*) and bacteria (e.g., *Burkholderiaceae*) were identified in the lignin-enriched cultures. All the microbially solubilized lignin products were likely to stimulate plant root elongation in the order selected white-rot fungi > microbial consortia > antibacterial agent-treated microbial consortia. Overall, this study supports the idea that microbial transformation of lignin can contribute to the formation of biologically active organic matter.

**IMPORTANCE** Structurally stable humic substances (HSs) in soils are tightly associated with soil fertility, and it is thus important to understand how soil HSs are naturally formed. It is believed that microbial metabolism on plant matter contributes to natural humification, but detailed microbial species and their metabolisms inducing humic functionality (e.g., direct plant stimulation) need to be further investigated. Our findings clearly support that microbial metabolites of lignin could contribute to the formation of biologically active humus. This research direction appears to be meaningful not only for figuring out the natural processes, but also for confirming natural microbial resources useful for artificial humification that can be linked to the development of high-quality soil amendments.

## INTRODUCTION

Humic substances (HSs) are chromogenic polymers whose small components are supramolecularly assembled (1, 2). Depending on molecular weights and water solubility, HSs are classified into fulvic acids, humic acids, and humins (2). Although humic structures are very complicated due to the compositional heterogeneity and the structural irregularity, it has been demonstrated that the presence of HSs leads to enhance soil fertility (3–5). The potential applicability of HSs as a soil conditioner was revealed by confirming their capacity to aggregate soil particles, thereby stabilizing soil structures (6). Another HS beneficial action on crops derives from their direct plant stimulation, wherein some humic components penetrate plant root interiors, followed by the induction of several gene and protein expressions of plants (7–9). These expression changes tend to coincide with different plant phenotypes. For instance, plant seed germination is accelerated in the presence of HSs, and this could be ascribed to the activation of auxin-like plant hormone pathways (10). Salt-induced abiotic stresses to plants are also mitigated with HSs, wherein the delayed degradation of plant high-affinity K+ transporter 1 is induced (9). Low-molecular-weight components of HSs appear to be involved in the direct plant stimulation owing to their high water solubility and plant root penetration capacity (2).

The origin of HSs in the environments remains to be fully described, but structural evidence to underpin similarities between HSs and plant lignin has been continuously reported. Lignin-derived organic moieties (e.g., phenolic and carboxylic acid structures) are indeed widespread in HSs (2, 11). The inherent recalcitrance of lignin combined with mineral association contributes to the supply of stabilized soil organic matter (12). Interestingly, Jiang et al. proved that lignin degradation could be accompanied by HS formation during composting (13). In addition, the positive correlation between HS production and populations of laccase-producing microbes is in accordance with the fact that microbial lignin metabolism results in the release of metabolic end-products that are recalcitrant and thus persistent in soils. It is also noticeable that new organic functional groups that are not found in lignin, but are found in HSs, can be formed by microbial metabolism of lignin (14).

The contribution of plant lignin to soil humification is presumably significant, but it is still questionable whether microbial metabolism of lignin is essential for displaying plant-stimulatory activities of HSs. Microbial metabolites of lignin have been investigated, and key enzymatic reactions are suggested (15–17), but to our best knowledge, it appears that no clear connection between microbial metabolism-driven lignin structural modifications and plant-stimulatory activities has so far been reported. Since direct plant stimulation capacity is one of the main features of soil HSs, the connection must be proven for fully rationalizing the notion that lignin is the origin of HSs. Moreover, structural complexities of HSs make it hard to determine crucial structural changes of lignin resulting in humification. In this regard, it would be more acceptable to evaluate unique HS functionalities as a criterion of lignin humification.

White-rot fungi are renowned for degrading lignin by employing ligninolytic exoenzymes (18, 19), but bacteria incapable of direct lignin metabolism would likely be involved in the transformation processes by using fungal lignin metabolites as substrates. In other words, microbial consortia would play an important role in transforming lignin architecture and, in turn, humification in soils. In fact, the increase of bacterial population along with dead wood decomposition was experimentally identified (20). Depolymerized lignin products are also found to be metabolized with bacterial species (21, 22). Thus, which types of environmental microbes are necessary to facilitate transformation of lignin toward biologically functional HSs needs to be evaluated.

The aim of this study was to identify the essential microbial activities needed for biologically functional humification of plant lignin, thus elucidating the causal factors of HS plant-stimulatory properties. Selected white-rot fungi (i.e., Ganoderma lucidum and Irpex lacteus) and microbial consortia derived from plant litters and mountain soils were recruited to metabolize plant lignin. A potato dextrose broth mixed with lignin was used to proliferate the selected fungi and to enrich the microbial consortia, and in the enrichment of environmental microbes, well-known antibiotics (i.e., erythromycin and chloramphenicol) were also treated to hamper bacterial proliferation and metabolism. Microbial community analyses before and after the enrichment were conducted based on next-generation sequences (NGSs) of DNAs extracted from the environmental samples (i.e., plant litters and mountain soils) and the enrichment cultures.

After the cultivations, solubilized lignin metabolites were harvested in the supernatants, followed by analyses of their polymeric sizes, radical-scavenging capacity, chromogenic features, mass spectrometry, protein quantification, and total phenolic contents. Control lignin obtained from the same broths but not inoculated was also used for the comparison. In the case of the selected fungal cultures, additional analyses of exoenzyme profiling and solid-state ^13^C nuclear magnetic resonance (NMR) were further conducted. Finally, lettuce growth was monitored in the presence of the lignin variants and compared with that of the control lignin, thereby assessing the enhancement of crop-stimulatory activity by microbial lignin metabolism. Leonardite humic acids used for agronomical purposes were also used for the comparison.

## RESULTS AND DISCUSSION

### Lignin-derived products by selected white-rot fungi.

**FIG 1:**
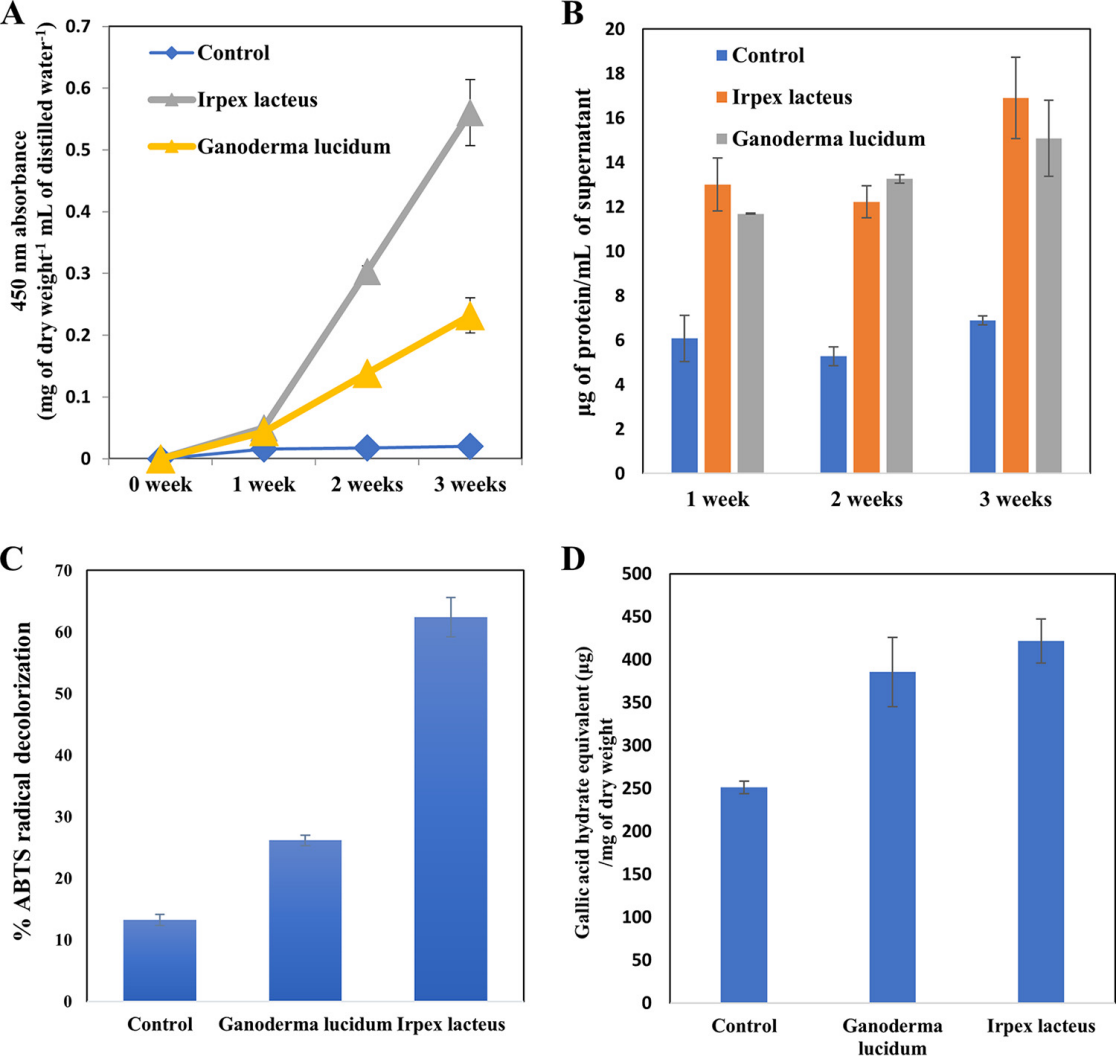
(A) Visible light absorbance (450 nm); (B) Bradford assay-based protein quantification; (C) ABTS decolorization capacities; (D) total phenolic contents of solubilized products in noninoculated control and fungal cultures. Average and standard deviation (*n* = 2) are shown. Another experimental set (*n* = 3) showed the similar results.

Brown colors in the supernatant parts of the liquid cultures of selected white-rot fungi (i.e., *G. lucidum* and *I. lacteus*) were observed with a naked eye, while relatively pale brown colors were seen in those of the noninoculated control cultures (data not shown), suggesting that more lignin-derived components are dissolved with fungal metabolism. Chromogenic properties of the supernatant parts were quantitatively assessed by measuring the extent of visible light absorbance, and as shown in Fig. 1A, the absorbance at 450 nm per unit solid content of each supernatant significantly increased with increasing incubation times. This result could be ascribed to the higher solubilization of brown-colored lignin with fungal metabolisms. To confirm whether any chromogenic materials are synthesized by the fungi without lignin, the supernatants from fungi alone were also compared at 450 nm. As shown in Fig. S1 in the supplemental material, the absorbance intensity was much less than for fungi in combination with lignin, indicating that microbial lignin solubilization is the main factor to increase the absorbance intensity.

To further explore this possibility, 2,2′-azino-bis(3-ethylbenzothiazoline-6-sulfonic acid) diammonium salt (ABTS) radical decolorization capacities and total phenolic contents of the supernatants were also evaluated, because the presence of products derived from lignin exhibiting polyphenolic moieties would likely be positively related to the two kinds of properties (23). As seen in Fig. 1, similar patterns with the visible absorbance were recorded in both ABTS radical decolorization and total phenolic contents, suggesting that lignin-derived products are accumulated in the supernatants with the fungal cultivations. Then we also quantified exoenzymes of the fungi by using a Bradford assay to confirm the possible involvement of fungal metabolisms in the lignin solubilization. In general, the quantity of enzymes secreted was in line with the visible absorbance with incubation times (Fig. 1).

To profile the detailed exoenzyme functions, we then analyzed proteomics by separating proteins from the supernatants. As listed in Tables 1 and 2, nonspecific oxidases such as peroxidase and Mn-dependent peroxidase were detected, suggesting that lignin is oxidized by these enzymes, thus enhancing their solubility by increasing oxygen-based functional groups and decreasing their molecular weights. In fact, it has been considered that these peroxidases are crucial for lignin biodegradation of white-rot fungi (15). Copper radical oxidases mainly capable of oxidizing alcohol groups were also detected in the both white-rot fungi employed. As Daou et al. suggested that this group of enzymes plays an important role in lignin structural changes by recognizing lignin phenolics as their substrates (24), they are likely to induce lignin solubilization by reacting with lignin phenolics. In addition, the catalytic action of copper radical oxidases results in the production of hydrogen peroxide, which is in turn used as an electron acceptor of lignin-degrading peroxidases (25). Other protein- and sugar-related enzymes identified would be due to the cosupply of potato containing proteins and carbohydrates to the fungal cultures (26, 27).

**TABLE 1:** Proteomic analysis of exoenzymes from *I. lacteus* fungal cultures^*a*^

Accession no.	Proteins identified	Protein content (mol %)
A0A0C9W4N5	Unplaced genomic scaffold scaffold_28, whole-genome shotgun sequence	0.23 ± 0.07
Q8LW55	Ribonuclease T2	3.2 ± 1.55
B0CNY1	Predicted protein	0.67 ± 0.27
P17576	Polyporopepsin	9.37 ± 4.88
P87212	Peroxidase	7.75 ± 5.23
A0A0P0I676	Mitochondrial choline dehydrogenase	5.03 ± 1.39
V5ND37	Metallopeptidase 1	0.59 ± 0.24
A0A0P0HVK7	Manganese peroxidase 3 (fragment)	3.97 ± 1.68
A0A0P0HPB0	Manganese peroxidase 3	6.24 ± 3.86
UPI000440B7AC	Hypothetical protein STEHIDRAFT_156755	0.87 ± 0.38
UPI0004407FDA	Hypothetical protein FOMMEDRAFT_141224	0.5 ± 0.37
A0A060SCW9	Glycoside hydrolase family 74/carbohydrate-binding module family 1 protein	0.23 ± 0.07
R7RWJ3	Glycoside hydrolase family 71 protein	0.47 ± 0.15
A0A0C3C4R3	Glycoside hydrolase family 3 protein	0.8 ± 0.06
S7S113	Glutaminase GtaA	0.28 ± 0.14
UPI00046216D2	Glucoamylase G2	0.63 ± 0.22
UPI0004415A45	Glucoamylase	0.93 ± 0.65
Q75NB5	Glucanase	4.15 ± 1.82
Q9Y724	Glucanase	0.98 ± 0.1
U6NLC6	Glucanase	0.44 ± 0.19
UPI000441823B	Family S53 protease	0.9 ± 0.08
UPI0004416B61	Endopeptidase	1.67 ± 0.47
Q5W7K4	Endoglucanase	1.48 ± 0.21
UPI0004622D89	DUF1793-domain-containing protein	0.4 ± 0.03
Q0ZKA4	Copper radical oxidase	0.29 ± 0.01
A0A0P0HFU3	Choline dehydrogenase	1.45 ± 0.17
A0A0P0I834	Choline dehydrogenase	0.31 ± 0.1
A0A060SZQ9	Carboxypeptidase	0.44 ± 0.19
M2R432	Carboxypeptidase	0.4 ± 0.03
A0A0C3NIQ6	Carboxylic ester hydrolase	0.75 ± 0.29
R7SY09	Carboxylic ester hydrolase	0.36 ± 0.17
G3XKT3	Aspartic protease	1.79 ± 1.51
UPI000440F5C0	Aspartic peptidase A1	0.52 ± 0.22
W4KAT8	Aspartic peptidase	0.39 ± 0.12
R7SSF9	Alpha-1,2-mannosidase	0.75 ± 0.29
UPI000444A5EF	Alpha/beta-hydrolase	0.36 ± 0.17

a The mol % (i.e., each emPAI/sum of emPAI × 100) and standard deviation (*n* = 2) are shown. Proteins detected in both duplicates are listed based on UniRef100 database (www.uniprot.org).

**TABLE 2:** Proteomic analysis of exoenzymes from *G. lucidum* fungal cultures^*a*^

Accession no.	Proteins identified	Protein content (mol %)
A0A0H2SDZ6	Tripeptidyl peptidase A	0.59 ± 0.33
Q8LW55	Ribonuclease T2	2.2 ± 0.27
P87212	Peroxidase	0.94 ± 0.08
A0A0P0I676	Mitochondrial choline dehydrogenase	4.73 ± 0.55
A0A0P0HVJ9	Manganese peroxidase 3 (fragment)	5.87 ± 2.8
UPI0004407FDA	Hypothetical protein FOMMEDRAFT_141224	1.44 ± 0.16
A0A067QAQ4	Glycoside hydrolase family 5 protein	2.08 ± 0.68
UPI0004415A45	Glucoamylase	0.41 ± 0.07
UPI000441823B	Family S53 protease	1.53 ± 0.22
R7SYU3	Endopeptidase	3.92 ± 0.53
Q0ZKA4	Copper radical oxidase	1.3 ± 0.42
A0A060SZQ9	Carboxypeptidase	0.5 ± 0.07
M2R432	Carboxypeptidase	1.03 ± 0.09
A0A0C9V748	Carboxypeptidase	2.21 ± 0.62
A0A0C3NIQ6	Carboxylic ester hydrolase	0.63 ± 0.24
G3XKT3	Aspartic protease	1.17 ± 0.15
UPI000440F5C0	Aspartic peptidase A1	1.17 ± 0.15
R7SSF9	Alpha-1,2-mannosidase	1.8 ± 0.17

a The mol % (i.e., each emPAI/sum of emPAI × 100) and standard deviation (*n* = 2) are shown. Proteins detected in both duplicates are listed based on UniRef100 database (www.uniprot.org).

### Structural elucidation of lignin-derived products by selected fungi.

**FIG 2:**
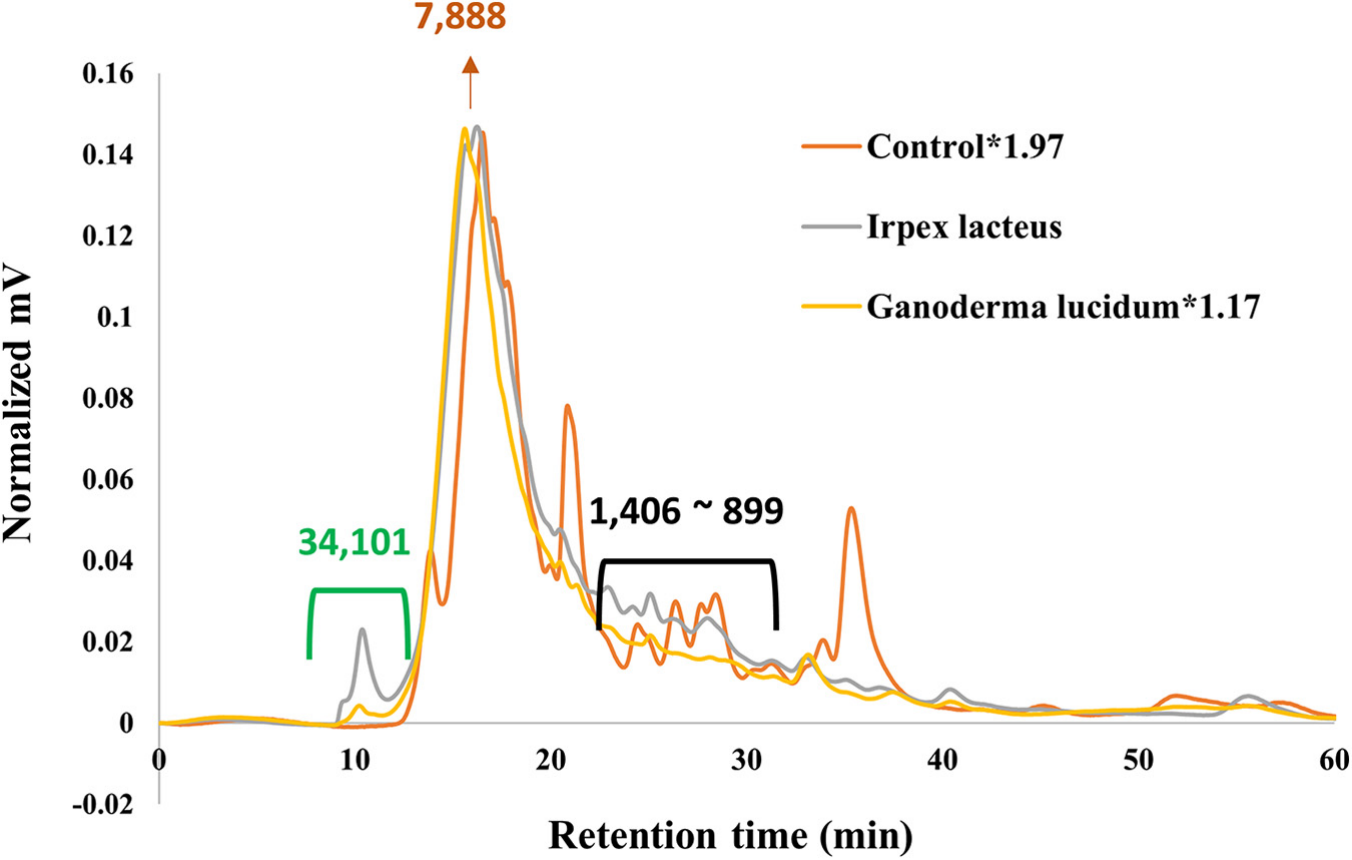
Size-exclusion chromatography of solubilized products in noninoculated control and fungal cultures. The green bracket indicates newly formed peaks with fungal metabolisms, and the black one indicates lignin-related peaks of the control.

**FIG 3:**
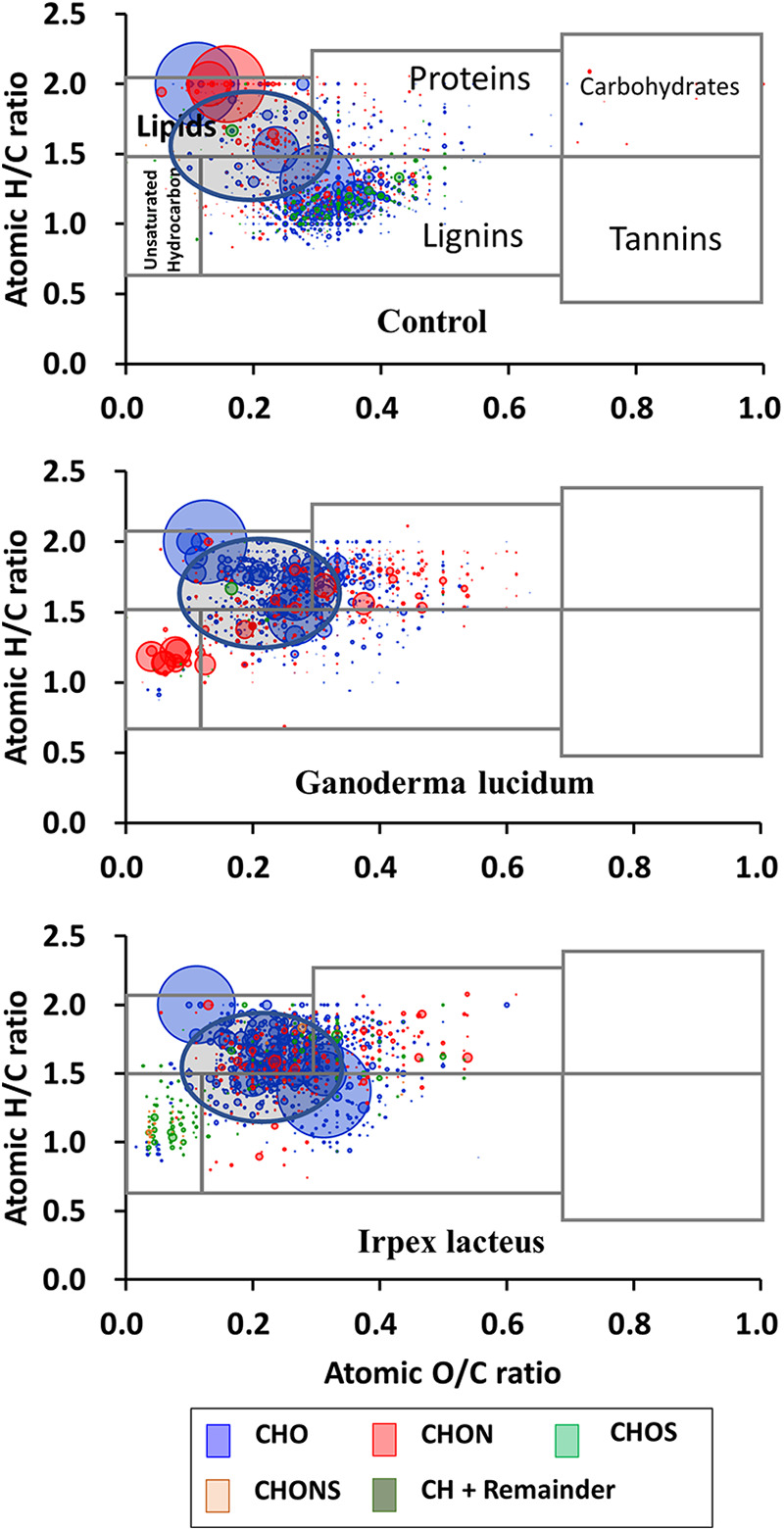
Van Krevelen plots showing the distribution of chemical classes based on the atomic H/C and O/C ratios of the assigned molecular formulas in supernatant components of noninoculated control and fungal cultures.

Size exclusion chromatography (SEC) and ultrahigh resolution Fourier-transform ion cyclotron resonance (FT-ICR) mass spectrometry were used to evaluate structural changes of lignin in more detail as shown in Fig. 2 and 3, respectively. Several sharp peaks showing fewer molecular weight (MWs) than the major peak corresponding to approximately a molecular weight of 8,000 were evident in SEC of the control (i.e., the lignin-containing broths that were not inoculated with the fungi), while the fungal cultures displayed one major peak near 8,000 MW that was dragged to lower MWs and a new peak showing a higher MW (i.e., 34,101 MW). By the comparison with SEC of the potato broth alone (data not shown), we concluded that peaks of the control ranging from 1,406 to 899 MW derived from lignin alone. Fungal metabolic pathways presumably oxidize them, thus allowing for their disappearance. The appearance of a new peak (i.e., 34,101 MW) in the fungal cultures would be attributable to solubilization of solid lignin by fungal metabolism. The overall absorbance at 280 nm in the SECs of the fungal cultures was higher than that of the control, indicating that more UV-absorbing products are solubilized with fungal metabolism. Given the multiple aromaticity of lignin capable of absorbing UV light ranges, the products would be from the solubilization of solid lignin.

The assignment of putative molecular formulas based on the mass spectrometric measurements visualized more significant changes in lignin before and after the metabolism. Molecules both composing carbon (C), hydrogen (H), and oxygen (O) and fittable to the lignin class of molar H/C and O/C ratios are highly likely to be lignin components (Fig. 3). The molecules in the control presumably derive from the soluble part of lignin initially supplied. Interestingly, a significant portion of them was molecularly transformed with fungal metabolism, thus exhibiting higher H/C ratios (circled regions in Fig. 3). This would be caused by the benzene ring opening, thus transforming aromatic groups into aliphatic groups. It has been similarly suggested that fungi are able to open benzene rings of lignin (14). Quinone-involved redox cycling by white-rot fungi is linked to the production of hydroxyl radicals capable of oxidizing aromatic compounds, which could be further involved in the ring opening (28). The peroxidases detected (Tables 1 and 2) presumably act as phenol oxidizers, thus forming quinone structure. It is also noticeable that molecules both belonging to the protein region and showing the composition of carbon (C), hydrogen (H), oxygen (O), and nitrogen (N) increased with fungal metabolism, which could be attributable to the presence of exoenzymes.

**FIG 4:**
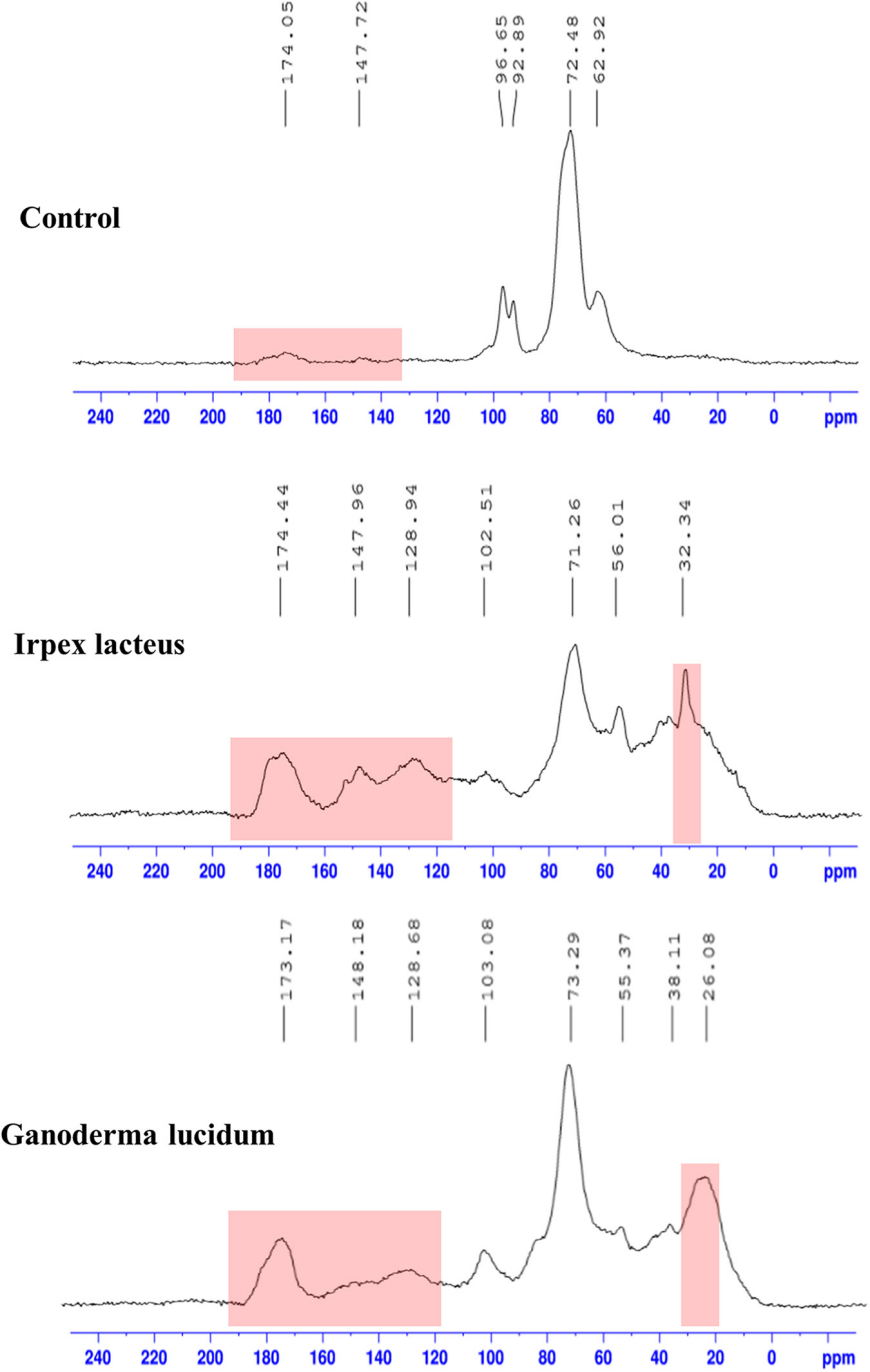
Solid-state ^13^C NMR spectra of solubilized products in noninoculated control and fungal cultures. Red boxes indicate peaks associated with lignin-related functional groups.

Solid-state ^13^C NMR revealed the structural features driven by fungal metabolism (Fig. 4). Major peaks shown in the control are very similar to those of starch, which could be ascribed to components of the potato broth (29). However, the small peaks near 174 and 147 ppm that may correspond to carboxylic and phenol groups, respectively (30), suggest that some lignin-derived components are present in the supernatant of the control, which is consistent with the result of the SEC and mass spectrometry results (Fig. 2 and 3). Interestingly, peaks near 32 ppm and corresponding to the alkyl group were newly formed with fungal metabolism (Fig. 4), and given the shift of the compositional molecules from lignin toward higher H/C ratios shown in Fig. 3, this shift would probably result from the benzene ring opening. Other interesting peaks enhanced with fungal metabolism are near 174 and 147 ppm (i.e., carboxylic and phenol groups, respectively). The intensity increases in these two peaks coincided with increasing peaks near 128 ppm, corresponding to aromatic rings, indicating that lignin-derived products accumulate in the supernatants of fungal cultures. Oxidative reactions by fungal exoenzymes may leave oxygen-based functional groups that are further involved in increasing hydrophilicity of lignin products. This interpretation is in line with the detection of carboxylic ester hydrolase in the supernatants of the fungal cultures (Tables 1 and 2) and a previous report showing the abundance of ester bonds by the *p*-coumaric acid moiety in plant lignin (31).

To further detail small lignin-derived phenolics in the supernatants, gas chromatography-mass spectrometry (GC-MS) was conducted (Fig. S2). Interestingly, one-benzene-ring-containing lignin-related aromatics such as vanillin and benzene acetic acid were not detected with the microbial cultivation. It would be related to phenol metabolism of white-rot fungi (32). Given that the GC column used is not able to detect oligomeric compounds, the chromatograms also indicate that the observed visible light colors with fungal growths could be attributable to the release of polyphenolic oligomers from insoluble lignin.

### Plant stimulation by lignin-derived products of selected fungi.

**FIG 5:**
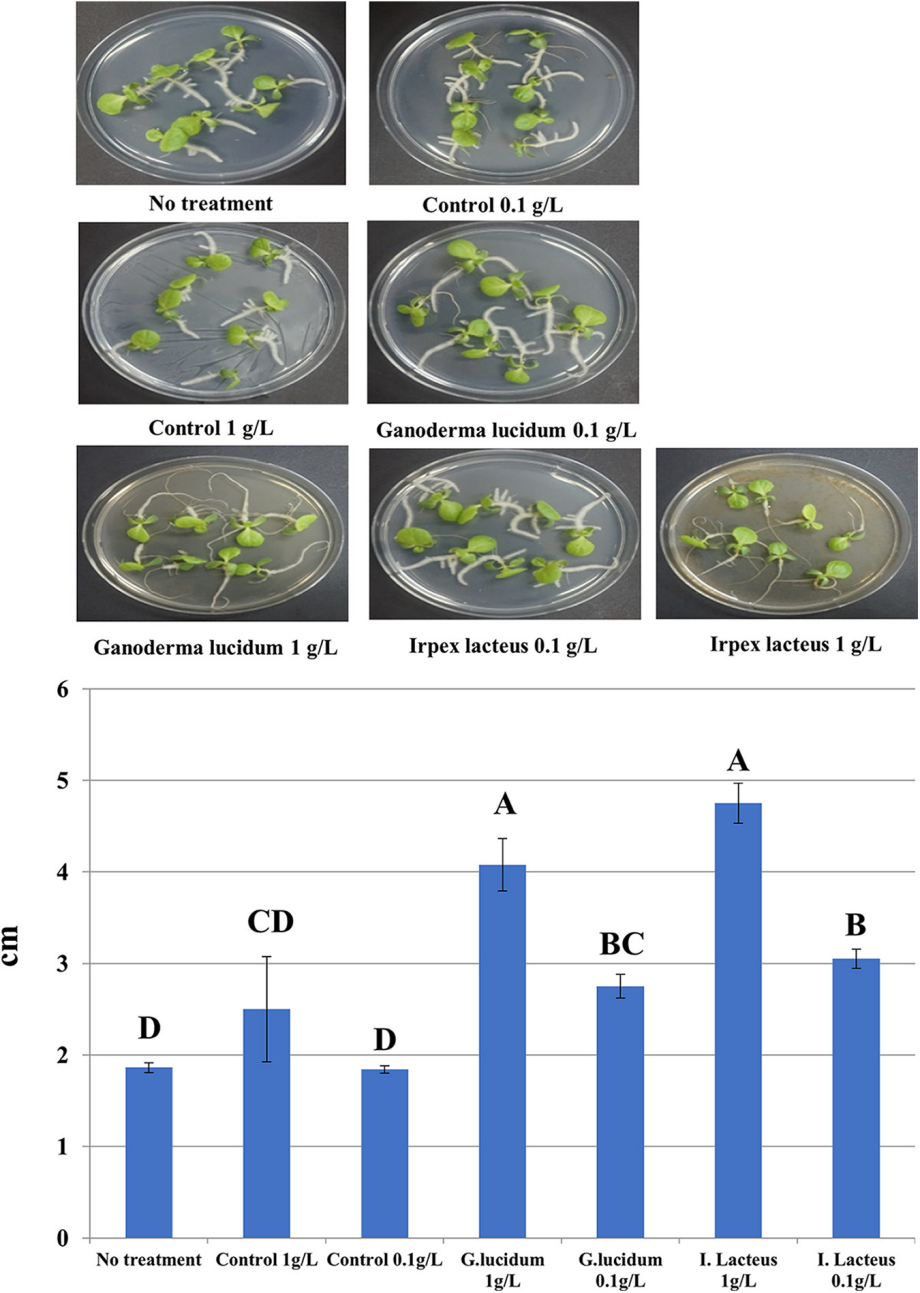
Root elongation of lettuce cultivated in MS agar mixed with solubilized products of noninoculated control and fungal cultures. Average and standard error (*n* = 14) are shown, and the data were statistically analyzed using one-way analysis of variance (ANOVA) and Duncan’s test (*P < *0.05).

We then tested whether the lignin solubilized by fungal metabolism is effective to stimulate plants. As shown in Fig. 5, lettuce root morphologies were significantly modified, while the control samples which were not affected by fungal metabolism exhibited no difference from the lettuces treated with nothing. In particular, the axial roots tended to grow more rapidly with the solubilized lignin products by fungal metabolism. Root growth-stimulatory actions of HSs have been well demonstrated (2, 10), and auxin-related plant hormone pathways appear to be involved in the stimulation (10). Structurally, phenolic and carboxylic groups in HSs play an important role in the stimulations (10) and seem to be present in the current fungal lignin-derived products. We then also compared the stimulatory activities with those of commercial humic acids that are used for agronomical purposes. There was a noticeable difference between the untreated and the humic-treated plant root growths. In addition, the magnitude of the root stimulation was almost comparable to that of lignin plus fungus inoculation (Fig. S3). Overall, these results indicate that fungal metabolism on plant lignin is involved in the production of biologically active organics.

### Use of microbial consortia for lignin metabolism.

**FIG 6:**
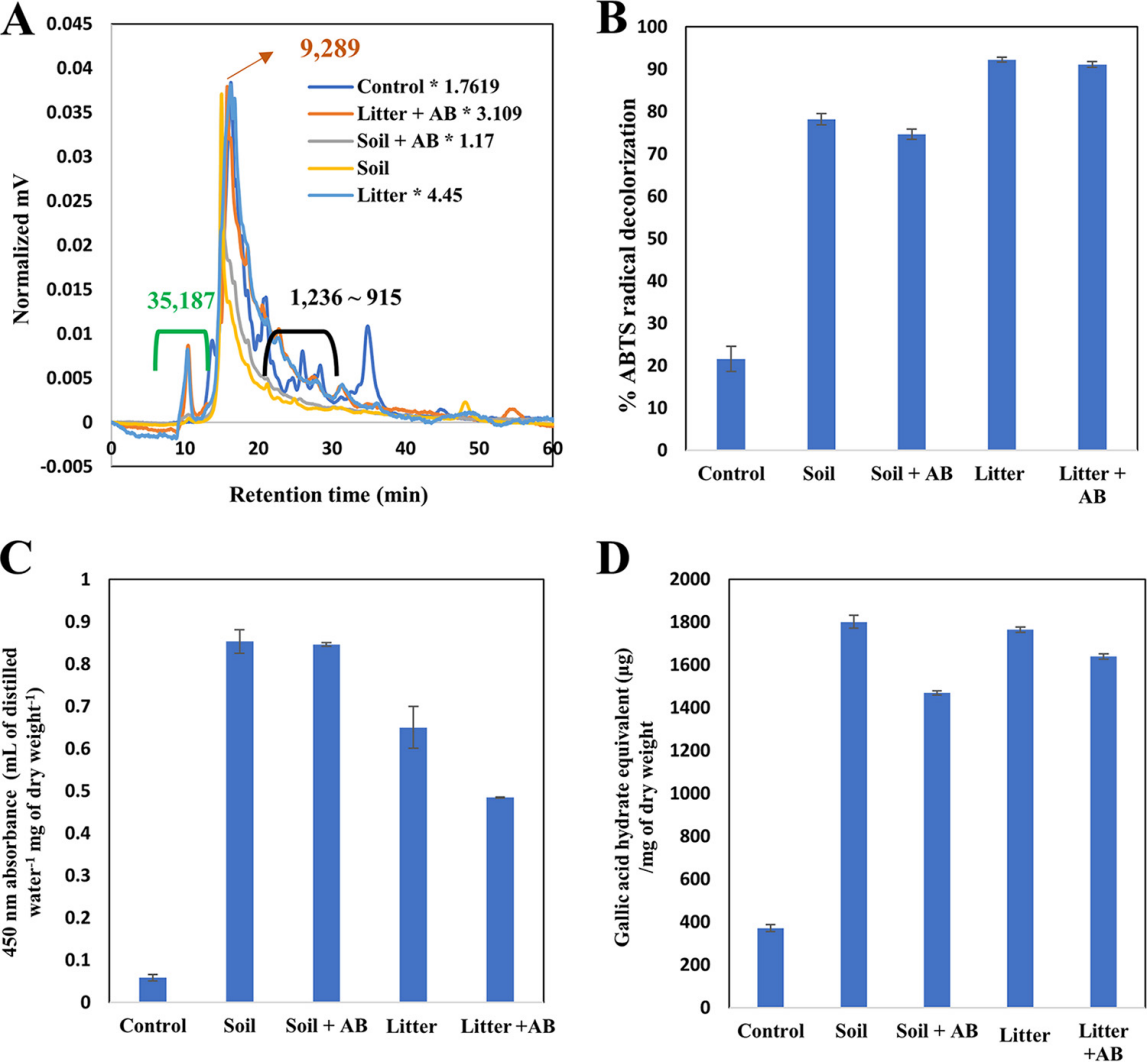
(A) Size-exclusion chromatography wherein the green bracket indicates newly formed peaks with microbial metabolisms, and the black one indicates lignin-related peaks in the control. (B) ABTS decolorization capacities, (C) visible light absorbance (450 nm), and (D) total phenolic contents of solubilized products of noninoculated control and microbial consortia. AB, antibacterial agents. Average and standard deviation (*n* = 2) are shown. Another experimental set (*n* = 3) for panels B, C, and D showed similar results.

Beyond the use of isolated white-rot fungi, we endeavored to study the role of environmental microbes for this phenomenon. The same potato broths containing lignin were used to enrich environmental microbes of plant litters and mountain soils. We also used antibacterial drugs (i.e., erythromycin and chloramphenicol) to confirm whether bacterial metabolism could aid in the functional humification. As shown in Fig. 6, very similar properties of all the supernatants with those of the selected fungal cultures (i.e., enhancement of chromogenic, radical-scavenging capacities and total phenolic contents) were observed with the enrichment of environmental microbes. These results imply that some of the microbes enable lignin metabolism, wherein solid lignin components are solubilized. In addition, bacterial metabolism was found not to be obligatory, considering the inability of the antibacterial drugs to hamper the lignin solubilization. It thus appeared that the fungal community is a sufficient condition to induce such structural changes in lignin (Fig. 6).

**FIG 7:**
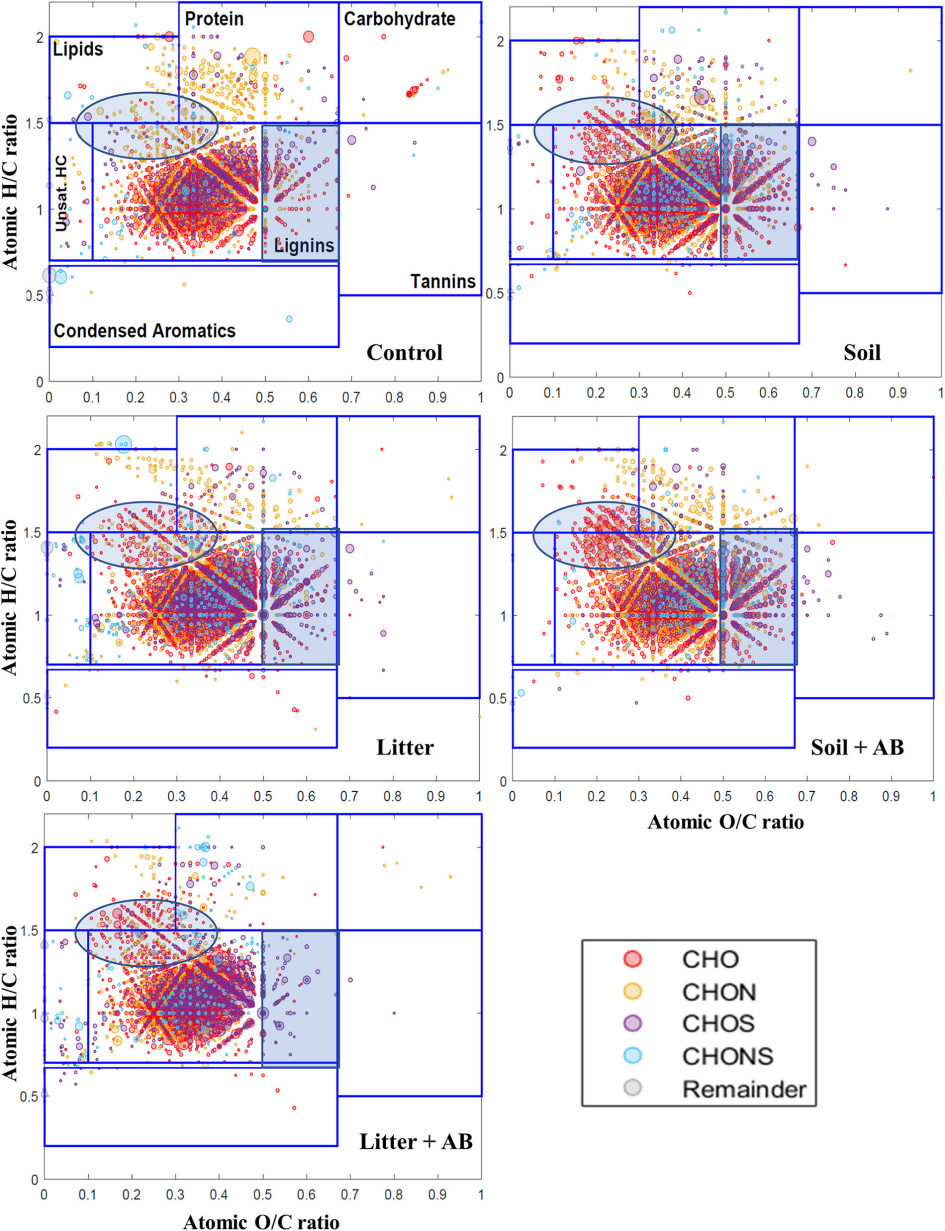
Van Krevelen plots showing the distribution of chemical classes based on the atomic H/C and O/C ratios of the assigned molecular formulas in supernatant components of noninoculated control and microbial consortia. AB, antibacterial agents.

Molecular composition assignments by the ultrahigh-resolution mass spectrometry clearly visualized the lignin transformation that is similar to that of the selected fungi. The molecules composing carbon (C), hydrogen (H), and oxygen (O) and also belonging to the lignin class shifted toward the range having higher H/C ratios (circled regions in Fig. 7). In addition, the movement toward higher O/C ratios was significantly observed, except for the litter treated with antibiotics (rectangular regions in Fig. 7). These changes could be caused by oxygenation of lignin and benzene ring opening. The presence of antibiotics hardly induces the changes in the distribution of molecules, and this raises the question of whether the enrichment of fungal species overwhelms bacterial metabolism in the given nutrient broth.

**FIG 8:**
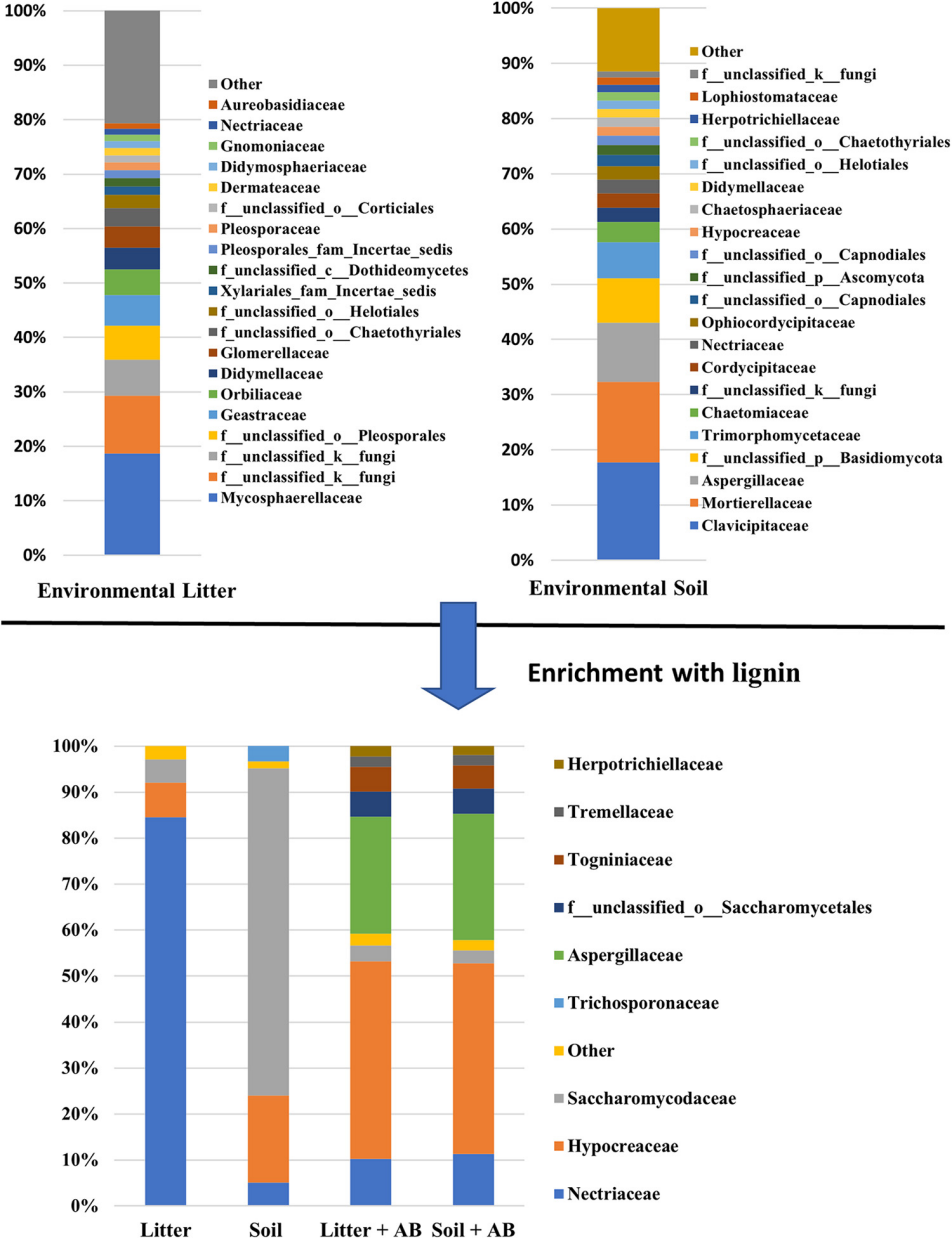
Relative abundance of fungal communities of environmental samples used for the inoculation (i.e., plant litters and mountain soils) and the enriched cultures at the family level. Taxa with an abundance of <1% are included in “other.” AB, antibacterial agents; environmental, real environmental samples.

The microbial enrichment patterns shown are based on next-generation sequencing (NGS)-based microbial community analyses whose rarefaction curves were saturated (Fig. S4), indicating that the communities profiled are representative of the cultures and the real environments. The significant decreases in the microbial diversity were recorded (Fig. 8, Fig. S5 and S6), meaning that carbon sources of the potato broth and lignin act as an enrichment pressure to help selected microbes flourish. Interestingly, different microbial families between plant litters and soils were enriched, which could be attributable to different microbial compositions of the inoculants. Nonetheless, *Burkholderiaceae* in bacteria and *Nectriaceae*, *Hypocreaceae*, and *Saccharomycodaceae* in fungi were commonly detected, suggesting that these could be important for lignin metabolisms. Indeed, *Burkholderiaceae* is renowned for aromatic degradation (33), and *Nectriaceae* and *Hypocreaceae* are also identified during natural wood decay (34, 35). Their detailed roles in lignin metabolism need to be further investigated by using culture-dependent techniques. It is also remarkable that the treatment of the antibiotics induced very similar fungal communities, irrespective of the kinds of inoculants (Fig. 8).

**FIG 9:**
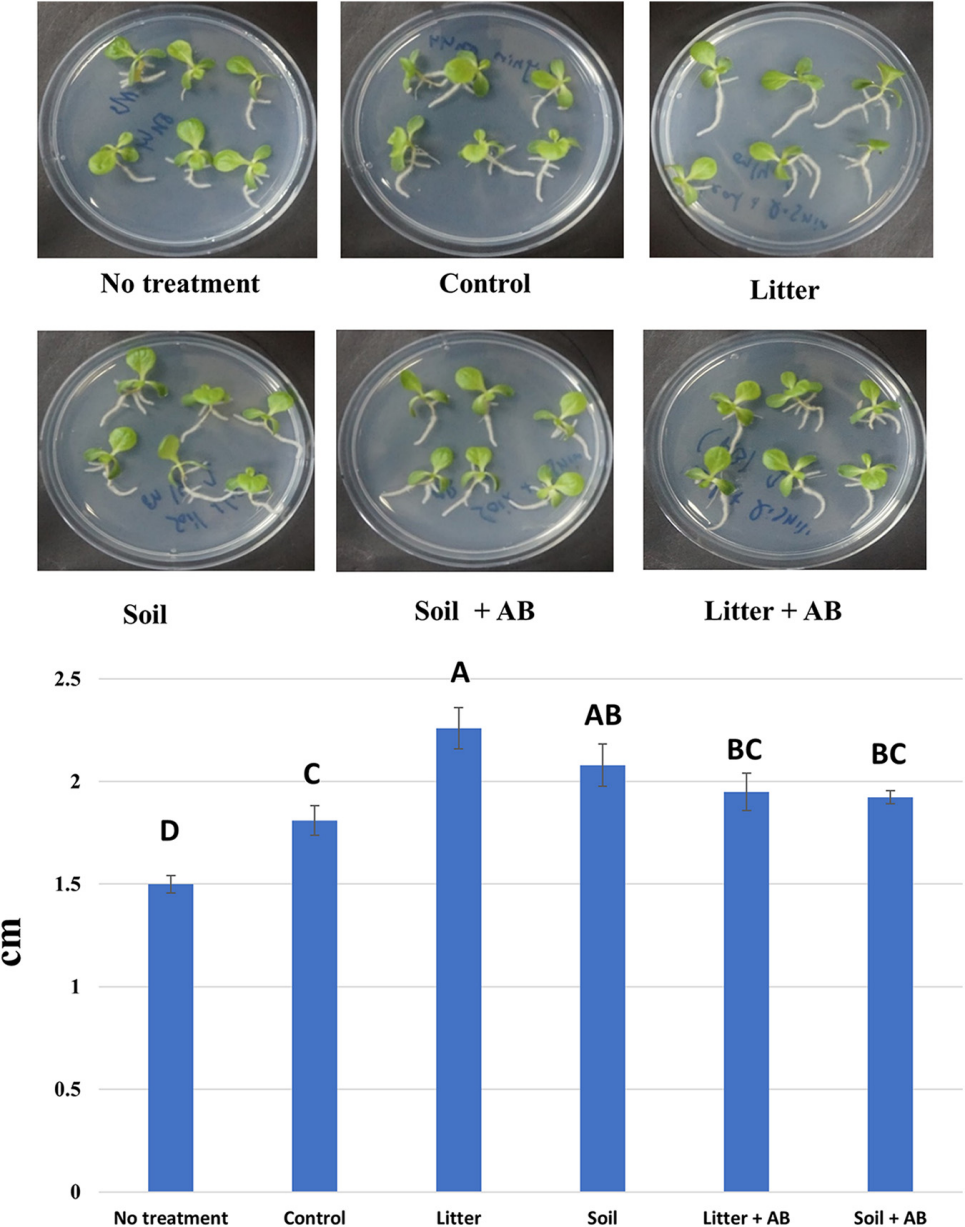
Root elongation of lettuce cultivated in MS agar mixed with solubilized products (0.1 g L^−1^) of noninoculated control and microbial consortia. Average and standard error (*n* = 18) are shown, and the data were statistically analyzed using one-way ANOVA and Duncan’s test (*P < *0.05). AB, antibacterial agents.

Finally, it was confirmed that the root growth is enhanced with the enrichment of environmental microbes. As shown in Fig. 9, the root lengths affected by lignin-derived products of the microbial consortia tended to increase compared with the control (i.e., lignin alone) and no treatment, although the extent of the increase appeared to be less than that by the selected fungal broths (Fig. 5). These results suggest that plant litters and mountain soils contain microbial species enabling the production of lignin metabolites that could potentially lead to the biologically functional humification of lignin. On the other hand, the treatments of the antibiotics appeared to result in less root-stimulatory activity (Fig. 9). This would be indicative of better lignin transformation efficiency by the cooperation between fungal and bacterial metabolism, which is supported by the fact that bacteria are coproliferated when wood is naturally decayed (20). HSs are known to stimulate plants in a various ways (2). Depending on the experimental situations, changes in the plant physiology of seed germination, abiotic stress responses, and aboveground biomass growth could be induced. Hence, further studies to assess such changes with the microbially transformed lignin products need to be performed.

Here, we confirmed that selected white-rot fungi and microbial consortia derived from plant litters and mountain soils are able to not only solubilize lignin but also endow lignin with plant-stimulatory activity (i.e., root elongation). The biologically functional lignin humification by white-rot fungi coincided with the secretion of ligninolytic enzymes such as peroxidase and Mn-dependent peroxidase, copper radical oxidases, and carboxylic ester hydrolase. The litters and soils were found to contain microbial species enabling lignin transformation connected to plant-stimulatory capacity, wherein bacterial metabolisms appeared not to be obligatory, and *Nectriaceae*, *Hypocreaceae*, and *Saccharomycodaceae* in fungi and *Burkholderiaceae* in bacteria were mainly enriched. Given that most studies linking microbial lignin transformation to humification do not consider how biological activities of HSs originated (14, 36, 37), this study provides new knowledge that microbial metabolism endows lignin with plant-stimulatory activity.

## MATERIALS AND METHODS

### Chemicals.

Kraft lignin (alkali), erythromycin, ammonium bicarbonate, iodoacetamide, Trametes versicolor laccase (≥0.5 U mg^−1^), and Folin-Ciocalteu phenol reagent were obtained from Sigma-Aldrich, while saccharose, sodium carbonate (99%), methanol (99.9%), sodium phosphate monobasic (99%), sodium phosphate dibasic (99%), ethyl acetate (high-performance liquid chromatography [HPLC] grade), and sodium azide (99%) were purchased from Daejung Chemicals (Siheung, Republic of Korea). Bradford assay solution, chloroform, and chloramphenicol were from Bio-Rad, Mallinckrodt and PhytoTech Labs, respectively. Bacto agar and potato dextrose broth were from BD Difco, while Murashige and Skooge (MS) medium was from KisanBIO (MBcell, Seoul, Republic of Korea). Agarose LE was purchased from GeorgiaChem. Gallic acid hydrate, ferulic acid and 2,2′-azino-bis(3-ethylbenzothiazoline-6-sulfonic acid) diammonium salt (ABTS) were from Tokyo Chemical Industry. Lettuce seeds (Lactuca sativa L., cv. “Yeoreum Jeokchima”) were obtained from Jeilseed (Seoul, Republic of Korea). Modified porcine trypsin (sequencing-grade) and trichloroacetic acid were obtained from Promega and Merck, respectively. Dithiothreitol and urea were from GE Healthcare, while acetone, acetonitrile, methanol for HPLC and formic acid were obtained from J.T.Baker. *G. lucidum* (Korean Agricultural Culture Collection [KACC] no. 42231) and *I. lacteus* (KACC no. 43133) were kindly donated by the National Institute of Agriculture Sciences (Jeonju, Republic of Korea). Plant litters and mountain soils were obtained from a small mountain near Gyeongsang National University (Jinju, Republic of Korea). Bovine serum albumin (biotechnology grade) was purchased from BioShop Canada, while poly(styrene sulfonate) standard materials were purchased from PSS Polymer (Maryland, USA). Leonardite-derived commercial humic acids were obtained from Mycsa AG (Texas, USA).

### White-rot fungi and microbial consortium cultivation for lignin biotransformation.

Two white-rot fungi (*G. lucidum* and *I. lacteus*) were maintained on potato dextrose agar. For the lignin transformation, lignin powder (0.4 g) was mixed with potato dextrose broth (100 mL), followed by autoclaving (121°C for 15 min). After the inoculation of the fungi from the agars, the broths were maintained for 4 weeks (160 rpm and 30°C), and solubilized lignin products were then harvested by centrifugation (4,000 rpm for 10 min). The supernatants were further subjected to freeze-drying, thereby obtaining powders of lignin metabolites. Tiny amounts of environmental samples (i.e., plant litter and mountain soil) were inoculated into the same broths with or without antibacterial agents (i.e., both erythromycin [1 mg] and chloramphenicol [1 mg]) and then incubated for 4 weeks (160 rpm and 30°C). The lignin metabolites were harvested the same way as the fungal cultures. Control powders were also obtained using the same harvest method of the lignin (0.4 g)-containing broths (100 mL of potato dextrose broth) that were incubated for 4 weeks (160 rpm and 30°C) without the microbial inoculations. Additionally, fungi alone without lignin addition were cultivated and then harvested in the same manner.

### Characterizations of solubilized lignin-derived products.

The lyophilized powders were dissolved in distilled water, and their absorbance at 450 nm was measured using a conventional UV-visible spectrophotometer. ABTS radicals were synthesized using *T. versicolor* laccase (5 mg) and ABTS (5 mg) in 5 mL of 100 mM sodium acetate buffer (pH 5.1). After 1 h, the blue-colored ABTS radical solution was ultrafiltered (Ultracell, 3 kDa; Amicon) to exclude the enzymes treated. Absorbance of the filtered solution at 420 nm was adjusted to 1.5 by diluting it with distilled water. After adding the broth-derived powders (300 μg of selected fungal cultures, 90 μg of microbial consortia, and the corresponding amounts of the controls per 1 mL of ABTS radical solution), the extent of ABTS radical decolorization was quantified by monitoring the absorbance decrease at 420 nm. Inherent absorbances of the powders in the solutions at 420 nm were compensated. Total phenolic contents were assessed as described previously (38). Briefly, the powders (15 mg of selected fungal cultures, 3 mg of microbial consortia, and the corresponding amounts of the controls) were dissolved in distilled water (2.25 mL) containing Folin-Ciocalteu phenol reagent (0.25 mL). After the solution was mixed for 5 min at room temperature, 2.5 mL of aqueous Na_2_CO_3_ (7%, wt/vol) solution was added to the solution and then incubated for 90 min at room temperature. The colorized solutions were then analyzed by measuring their absorbance at 550 nm. Inherent absorbances of the powders in the solutions at 550 nm were compensated, and gallic acid hydrate was used as a standard. The Bradford assay based on bovine serum albumin as a standard was used to assess protein concentrations of the supernatants (100 μL and 13,000 rpm for 10 min) of weekly harvested fungal broths.

HPLC (Waters 2695 system with a Waters 996 photodiode array detector) equipped with an SEC column (PolySep GFC-P3000 300 by 7.80 mm, Phenomenex) and a guard column (PolySep GFC-P 35 by 7.80 safety guard, Phenomenex) was used with a phosphate buffer solution (100 mM NaH_2_PO_4_, pH 6.5) containing sodium azide (4.6 mM NaN_3_) as the mobile phase. The broth-derived powders (10 mg) were dissolved in the mobile buffer (1 mL) before the HPLC injection (10 μL), and chromatograms based on 280 nm absorbance were normalized by using the highest peak for clarity. Polystyrene sulfonate showing 6,520, 14,900, and 145,000 molecular weights (MWs, Da) and ferulic acid were employed to make a calibration curve as performed previously (39). Solid-state ^13^C NMR was conducted at the Korean Basic Research Institute (KBSI) as described previously (10). Adamantane was used as an external standard.

Gas chromatography-mass spectrometry (GC-MS; GCMS-QP2020 NX, Shimadzu) equipped with a DB-WAX column (30 m by 0.25 mm; Agilent) was used to profile aromatics in the supernatants. The supernatants were treated with phosphoric acid for the acidification, followed by ethyl acetate-based liquid-liquid extraction. The solvent harvested was concentrated and then exchanged with acetone. The GC oven temperature was started at 50°C for 3 min and increased to 120°C (6°C min^−1^). Then, it reached up to 230°C (10°C min^−1^), followed by maintenance for 15 min. Electron impact was employed to fragmentize molecules with an *m/z* (45 to 800) scanning mode. Mass fragmentation patterns were compared with those of the NIST database.

### Microbial community profiling of microbial consortium cultivations.

Microbial consortium cultures after a 4-week cultivation and plant litters and mountain soils used for the inoculations were used to extract DNA using a DNA extraction kit (DNeasy PowerSoil Pro kit, Qiagen, Germany). NGS was then performed by Macrogen (Seoul, Republic of Korea), wherein DNA amplicons were made by using forward overhang adaptor sequence-added bacterial 16S ribosomal (341F: 5′-CCTACGGGNGGCWGCAG-3′ and 805R: 5′-GACTACHVGGGTATCTAATCC-3′) and fungal internal transcribed spacer (ITS) DNA regions (ITS3: 5′-GCATCGATGAAGAACGCAGC-3′ and ITS4: 5′-TCCTCCGCTTATTGATATGC-3′). Polymerase chain reactions (PCRs) (T100 thermal cycler, Bio-Rad) were conducted using Herculase II polymerase (Agilent, USA), followed by the purification of amplified products (AMPure XP Reagent, Beckman Coulter). Secondary PCRs were then performed with Herculase II polymerase (Agilent) and Nextera XT index primers. After the products were purified with AMPure XP reagent again, NGS was performed with a MiSeq 250 paired-end system. FLASH (v.1.2.11) was used to merge paired-end reads, and CD-HIT-OUT was employed to place tags with 97% identity in the same operational taxonomic units (OTUs). Representative OTUs showing higher than 85% of both query coverage and identity were taxonomically assigned based on reference databases (NCBI 16S rRNA for bacteria and UNITE+INSD for fungi) using BLAST (v.2.4.0). Statistical analyses of the microbial community were performed at https://www.microbiomeanalyst.ca/.

### FT-ICR-MS measurements and data processing.

The freeze-dried powders were dissolved in ultrapure water (1 mg/mL), which was then subjected to solid-phase extraction with an Oasis HLB cartridge (1 cc; Waters, Milford, MA) to remove inorganic constituents prior to FT-ICR mass spectrometric analysis. Briefly, the HLB cartridges were precleaned by passing 2 mL methanol through the cartridge and conditioned with 2 mL ultrapure water containing 0.1% formic acid. Then, 1 mL of the samples was passed through the HLB cartridge, followed by washing with 3 mL ultrapure water. After air drying, the organic components retained in the cartridges were eluted with 1 mL methanol containing 2% ammonium hydroxide solution.

The eluted samples dissolved in methanol with 2% ammonium hydroxide were analyzed using a 15T FT-ICR mass spectrometer (solariX XR system, Bruker Daltonics, Billerica, MA) equipped with a standard electrospray ionization interface in a negative ion mode, as previously described (40) with minor modification. The mass spectrometric spectra for the samples were recorded from *m/z* 150 to 1,200 with a transient size of 4 mega words in a negative ion mode and phase-corrected with the absorption mode, thereby obtaining a resolving power of 800,000 (full width at half maximum [FWHM] at *m/z* 400). External calibration was performed with quadratic regression using a NaTFA solution (100 μg/mL in methanol). The FT mass spectrometry data sets were subjected to further processing, including peak detection and recalibration using DataAnalysis (v.4.2, Bruker Daltonics) and elemental formula assignment using Composer (Sierra Analytics, Modesto, CA, USA). Briefly, the empirical molecular formulas were determined using only the masses of singly charged ions in the *m/z* range of 150 to 1,000 (allowing only formulas in the range C_1–100_H_1–200_O_1–60_N_0–4_S_0–1_,) and then the molecular formulas with assignment errors of >0.3 ppm were deemed to be too unreliable to be interpreted. The assigned chemical compositions were displayed based on the molar hydrogen-to-carbon (H/C) and oxygen-to-carbon (O/C) ratios on a van Krevelen diagram (41).

### Proteomic analyses of fungal exoenzymes.

After a 4-week cultivation (160 rpm at 30°C), 4 mL of the broths was used to harvest their supernatants (13,000 rpm for 10 min), followed by addition of 16 mL ethanol. Then, 4 mL of chloroform and 12 mL of distilled water were added, followed by a vigorous vortexing. The mixtures were then centrifuged (13,000 rpm for 3 min), and the pellets were washed with 16 mL of methanol. The pellets were finally lyophilized.

Further processes were conducted by Proteinworks (Daejeon, Republic of Korea). The pellets were dissolved in 1 mL of 8 M urea and vortexed for 1 h at 37°C. The supernatants were harvested after the centrifugation (13,000 rpm for 10 min), followed by mixing with 100 mM Tris-HCl (pH 6.8) as a 1:1 (vol/vol) ratio. Protein quantification was assessed based on bicinchoninic acid-based colorimetric detection. Trichloroacetic acid solution (10%) was made by adding 220 μg of proteins, followed by storage on ice for 30 min. The protein pellets harvested with the centrifugation (13,000 rpm for 10 min) were further washed with chilled acetone by a vigorous vortexing for 3 min. The reharvested pellets were dried at room temperature. Urea solution (5 μL of 5 M) was added to the pellets and then incubated for 1 h at room temperature. Trypsin solution (1 μg 5 μL^−1^) was treated after mixing the urea solution with 30 μL of 50 mM Tris-HCl buffer and incubated at 37°C for 16 h. Then, the enzyme was inactivated by heating up to 95°C for 3 min. Dithiothreitol was added up to 10 mM in the solutions and then incubated for 0.5 h at 56°C. Iodoacetamide was added up to 20 mM and then incubated for 1 h at room temperature without light. Desalting processes were based on a reverse-phase column (POROS R2, 20 μm bead size, PerSeptive Biosystems). The samples added with 20 μL of distilled water were loaded in the column. Formic acid (5% aqueous solution) and the solution containing 50% methanol, 49% distilled water, and 1% formic acid were sequentially used for the column washing and the elution, respectively. Finally, SpeedVac-derived samples were subjected to HPLC (UltiMate 3000 RSLCnano System, Dionex, Thermo Fischer Scientific, USA)-mass spectrometry (Q Exactive Plus hybrid quadrupole-orbitrap, Thermo Fischer Scientific) equipped with an Acclaim PepMap 100 C_18_ LC column (150 mm × 75 μm; Thermo Fischer Scientific) and a nano-electrospray ionization (ESI) source (Dionex, USA) at a flow rate of 300 nL min^−1^ with a gradient of 0.1% formic acid-containing acetonitrile from 0 to 65% for 180 min. Detailed protein search parameters are summarized in Table S1. An experimentally modified protein abundance index (emPAI) was used to quantify each protein concentration (42).

### Plant stimulation tests.

Lettuce seeds were surface-sterilized with a 30% bleaching solution (1.5% sodium hypochlorite and 0.02% Triton X-100) for 5 min and then washed five times with sterile distilled water and incubated for 2 days at 4°C before sowing. Seeds were germinated on MS medium (4.3 g L^−1^ MS, 30 g L^−1^ sucrose, pH 5.8, and 0.8% [wt/vol] agar) under a 16 h/8 h light/dark cycle at 23°C in a growth chamber. As soon as radicles appeared, the seedlings were transferred into new petri dishes containing the MS agar mixed with lignin-derived products of the fungal and microbial consortium cultures and the corresponding controls, followed by incubation in the same chamber. After 2 or 3 weeks, the primary root lengths were measured with a conventional ruler.

### Data availability.

The raw DNA sequencing data in this study have been deposited in the NCBI Sequence Read Archive (BioProject ID: PRJNA883187), and the raw mass spectrometric data and related protein list for fungal exoenzymes can be obtained at https://doi.org/10.6084/m9.figshare.21205691.v1.
