# Supplementary material for: Lignin Metabolism by Selected Fungi and Microbial Consortia for Plant Stimulation: Implications for Biologically Active Humus Genesis
Source: Microbiol Spectr. 2022 Oct 31;10(6):e02637-22. doi: 10.1128/spectrum.02637-22 (PMC9769858; doi:10.1128/spectrum.02637-22)

Supplementary material for

Lignin metabolism by selected fungi and microbial consortia for plant stimulation: Implications for biologically active humus genesis

Jalil Ur Rehman,[a,1] Eun-Nam Joe,[a,1] Ho Young Yoon,[a] Sumin Kwon,[a] Min Seung Oh,[a] Eun Ju Son,[d] Kyoung-Soon Jang,[d] and Jong-Rok Jeon[a,b,c]*

Addresses of Institutions:

[a]Division of Applied Life Science (BK21Plus), [b]Department of Agricultural Chemistry and Food Science & Technology & [c]IALS, Gyeongsang National University, Jinju 52828, Republic of Korea. [d]Bio-Chemical Analysis Team, Korea Basic Science Institute, Cheongju 28119, South Korea

[1]These two authors contributed equally to this work.

*Corresponding author: J.-R. Jeon (jrjeon@gnu.ac.kr)

Table S1. Search parameters used in the proteomic analyses of supernatants in non-inoculated control and fungal cultures.[a]

Search title	Control	Lac1	Lac2	Luc1	Luc2
Timestamp	2019-04-26T11:49:21Z	2019-04-26T13:44:46Z	2019-04-26T16:27:52Z	2019-04-26T20:14:18Z	2019-04-27T00:02:03Z
Peak list format	Mascot generic	Mascot generic	Mascot generic	Mascot generic	Mascot generic
Search type	MIS	MIS	MIS	MIS	MIS
Mascot version	2.4.1	2.4.1	2.4.1	2.4.1	2.4.1
Database	UniRef100	UniRef100	UniRef100	UniRef100	UniRef100
Fasta file	UniRef100_2016_03.fasta	UniRef100_2016_03.fasta	UniRef100_2016_03.fasta	UniRef100_2016_03.fasta	UniRef100_2016_03.fasta
Total sequences	76,839,110	76,839,110	76,839,110	76,839,110	76,839,110
Total residues	28,527,207,629	28,527,207,629	28,527,207,629	28,527,207,629	28,527,207,629
Sequences after taxonomy filter	4,640,607	4,640,607	4,640,607	4,640,607	4,640,607
Number of queries	8,937	14,790	19,385	26,465	26,821
matches	2	201	464	476	692
matchesDecoy	1	6	4	4	3
Taxonomy filter	. . . . Fungi	. . . . Fungi	. . . . Fungi	. . . . Fungi	. . . . Fungi
Maximum Missed Cleavages	2	2	2	2	2
Variable modifications	Carbamidomethyl (C),Oxidation (M)	Carbamidomethyl (C),Oxidation (M)	Carbamidomethyl (C),Oxidation (M)	Carbamidomethyl (C),Oxidation (M)	Carbamidomethyl (C),Oxidation (M)
Peptide Mass Tolerance	10	10	10	10	10
Peptide Mass Tolerance Units	ppm	ppm	ppm	ppm	ppm
Fragment Mass Tolerance	0.8	0.8	0.8	0.8	0.8
Fragment Mass Tolerance Units	Da	Da	Da	Da	Da
Mass values	Monoisotopic	Monoisotopic	Monoisotopic	Monoisotopic	Monoisotopic
Instrument type	ESI-TRAP	ESI-TRAP	ESI-TRAP	ESI-TRAP	ESI-TRAP
#13C	2	2	2	2	2
Significance threshold	0.01	0.01	0.01	0.01	0.01
FDR	50.000	2.985	0.862	0.840	0.434
Proteins	3	68	103	73	84
Sum of emPAI	0.22	10.48	16.54	10.95	11.30

[a]Abbreviations: Control, without inoculation; Lac, *Irpex lacteus*; Luc, *Ganoderma lucidum*; 1, duplicate 1; 2, duplicate 2

Figure S1. 450 nm absorbance of the supernatants after 4-week cultivation. Abbreviations: Lacteus, Irpex lacteus; Lucidum, Ganoderma lucidum. Average and standard deviation (n = 3) were shown

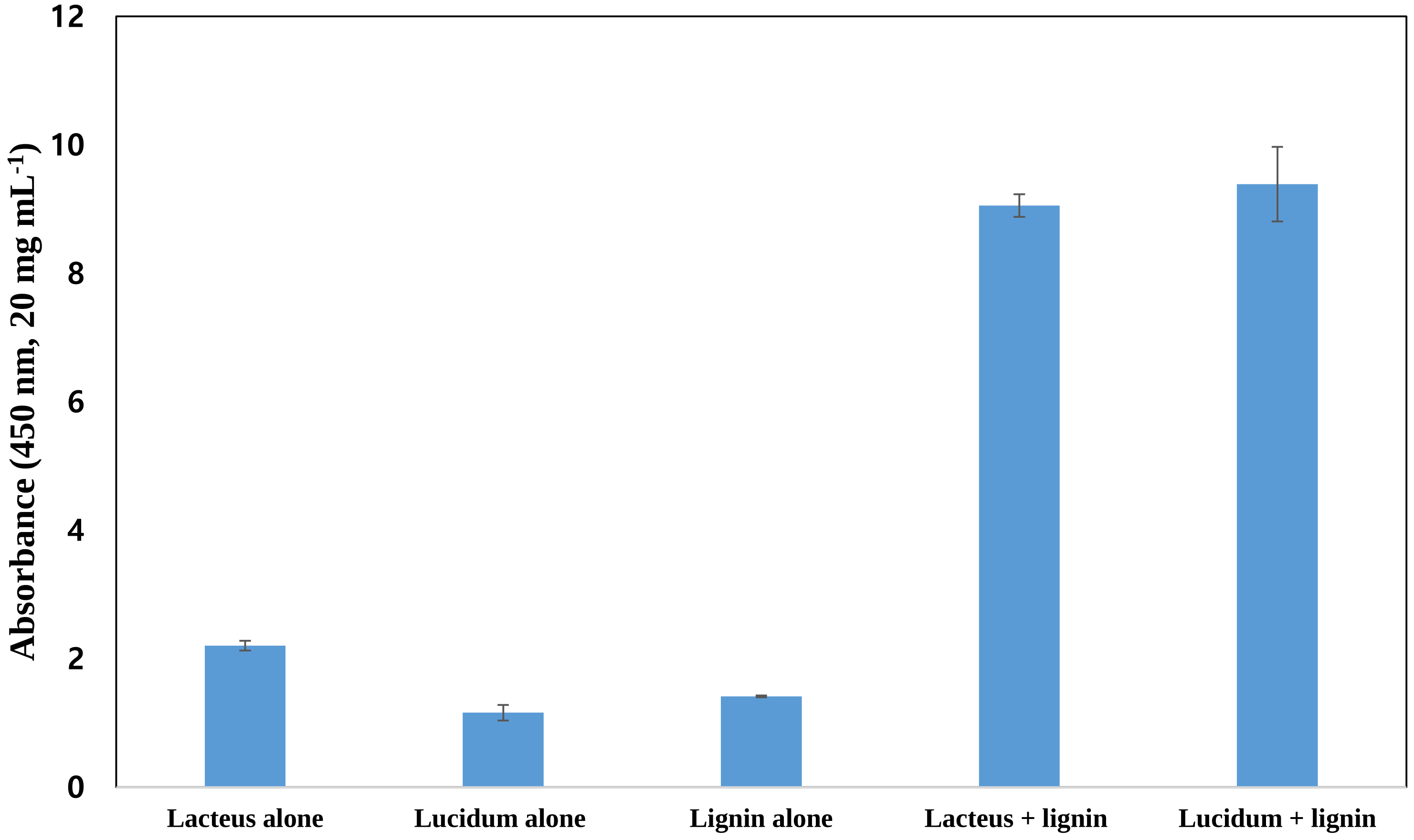

Figure S2. GC-MS profiling of low-molecular-weight aromatics in the supernatants of selected white-rot fungal cultures

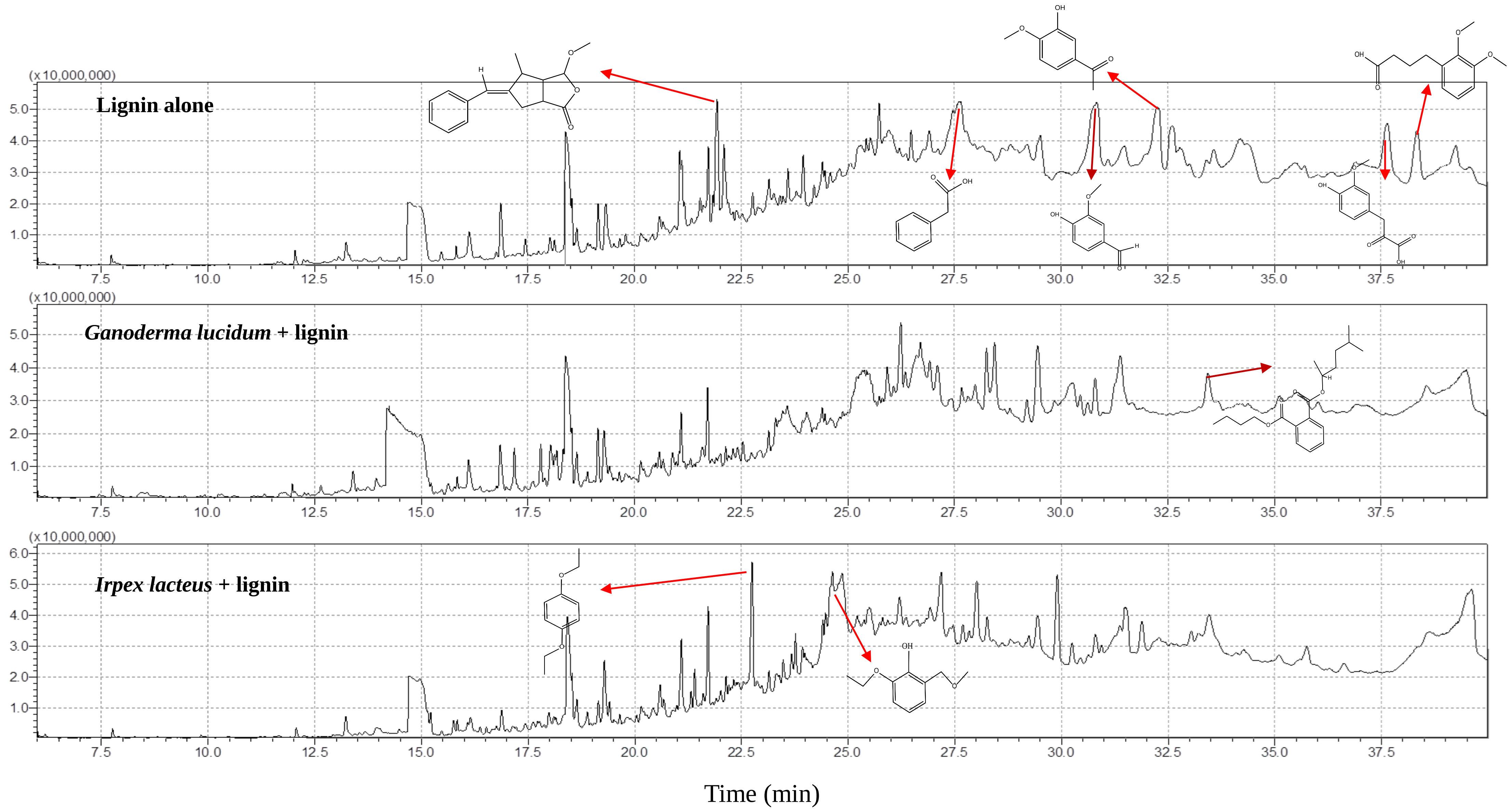

Figure S3. Root elongation of lettuce cultivated in MS agar mixed with solubilized products (0.1 g L^{-1}) of selected white-rot fungi and commercial humic acids. Abbreviations: Lacteus, Irpex lacteus; Lucidum, Ganoderma lucidum. Average and standard error (n = 10) were shown, and the data were statistically analyzed using one-way ANOVA and Duncan’s test (*p* < 0.05)

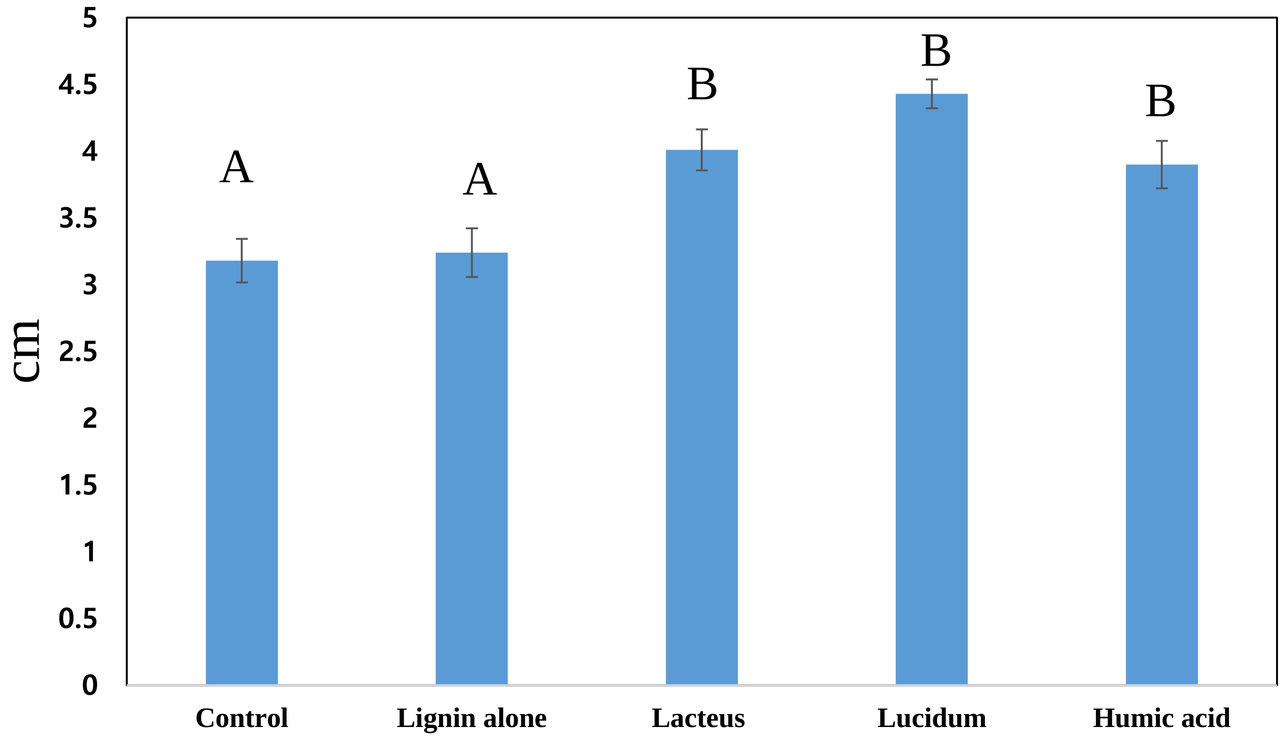

Figure S4. Rarefaction curves showing observed species richness in environmental samples used for the inoculation (*i.e.*, plant litters and mountain soils) and the enriched cultures. Abbreviation: Environment, real environmental sample.

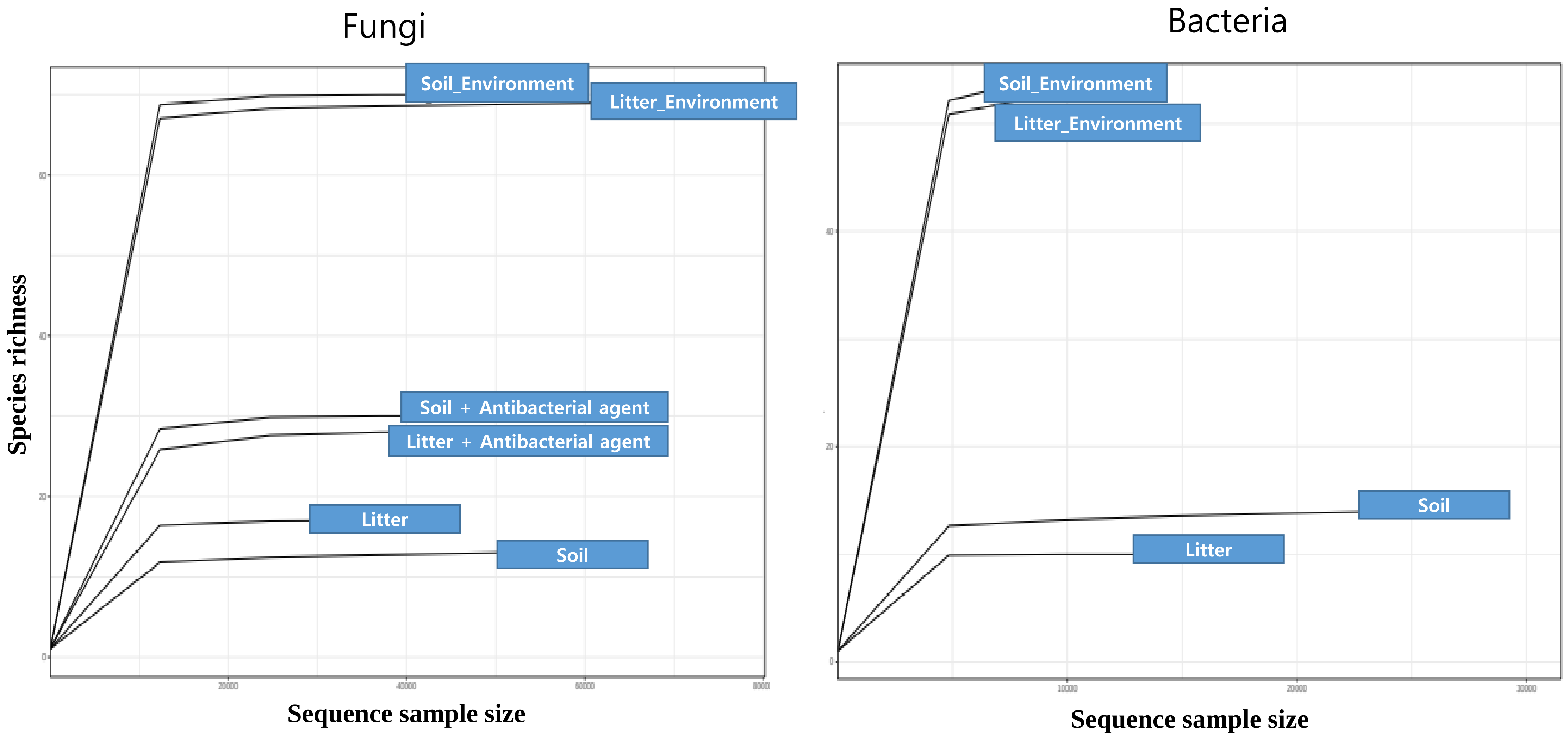

Figure S5. Alpha-diversity of environmental samples used for the inoculation (*i.e.*, plant litters and mountain soils) and the enriched cultures using Chao1 at Family level represented as boxplot. Abbreviation: AB, with antibacterial agents.

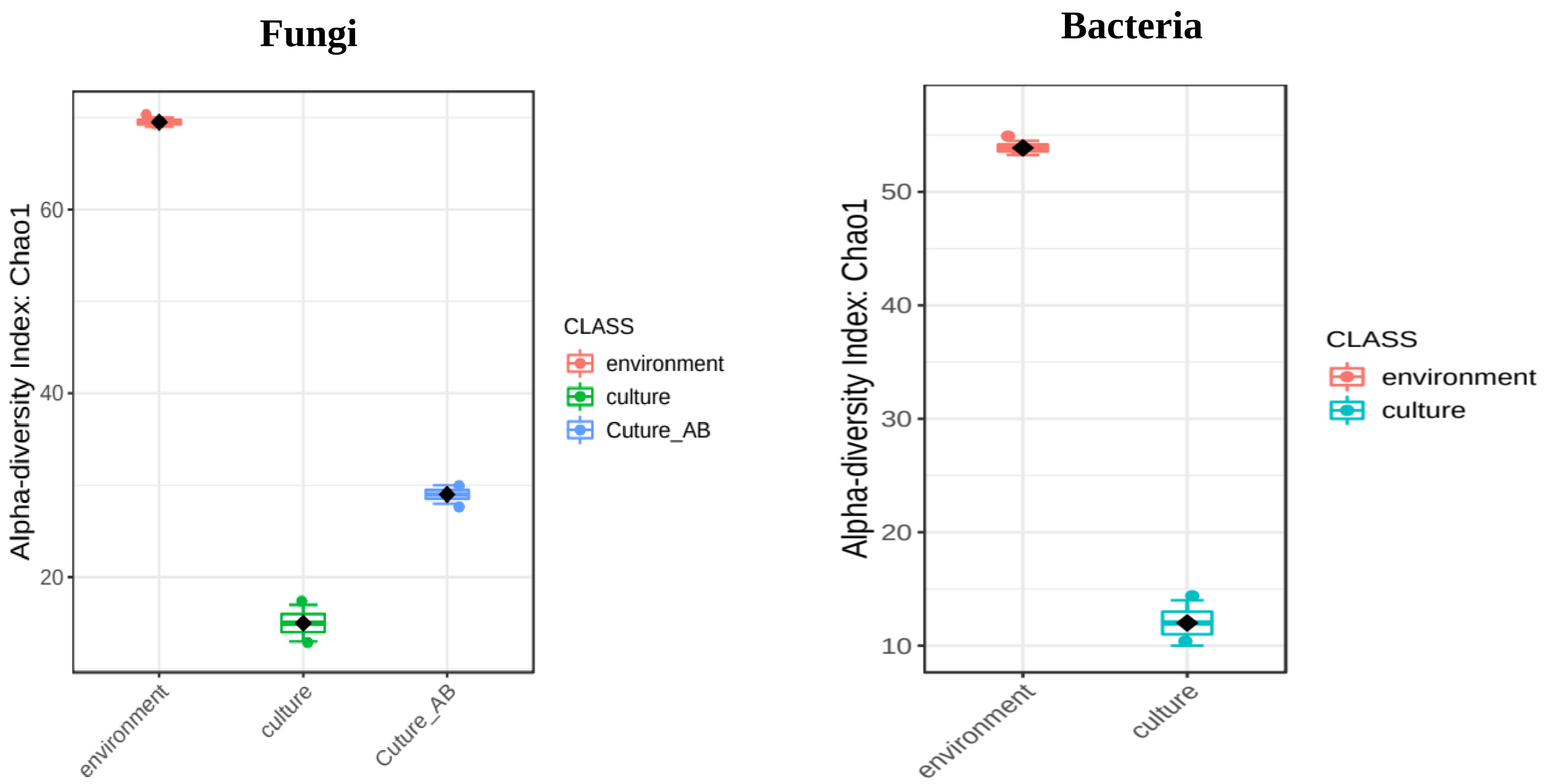

Figure S6. Relative abundance of bacterial communities of environmental samples used for the inoculation (*i.e.*, plant litters and mountain soils) and the enriched cultures at the family level. Abbreviation: environmental, real environmental samples. Taxa with an abundance < 1% are included in “other”.

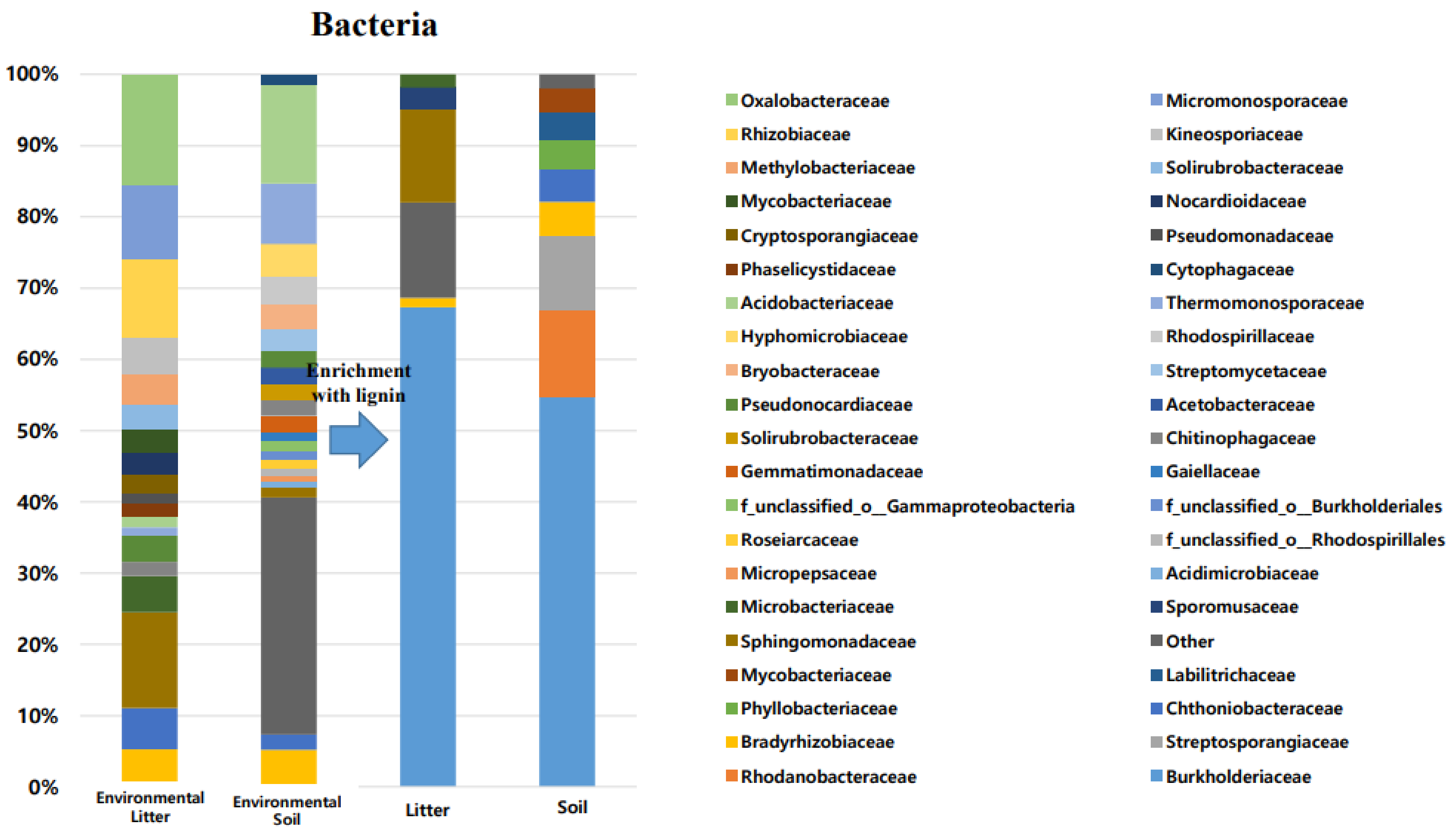

Supplement: Supplemental file 1 — Table S1 and Fig. S1 to S6. Download spectrum.02637-22-s0001.pdf, PDF file, 0.4 MB [file spectrum.02637-22-s0001.pdf]
